# Supplementary material for: Macrel: antimicrobial peptide screening in genomes and metagenomes
Source: PeerJ. 2020 Dec 18;8:e10555. doi: 10.7717/peerj.10555 (PMC7751412; doi:10.7717/peerj.10555)
Supplement: Supplemental Information 1 — The 22 features used for the classification of peptides into antimicrobial and hemolytic by the binary random forests implemented in Macrel are listed with the reference from which we took them as well as their relative importance. This "importance" refers to the number of times each feature was used during the random forest procedure. [file peerj-08-10555-s001.docx]

**Supplementary Table S1.** Features used in the MACREL classifiers.

| **Descriptor** | **Definition** | **References** | **Variable Importance to classification of** | |
| --- | --- | --- | --- | --- |
|  |  |  | **AMPs^a^** | **Hemolytic peptides^a^** |
| SA group 1 | Distribution parameter (CTDD) at first occurrence in the sequence using amino acids group 1 (A,L,F,C,G,I,V,W) clustered by solvent accessibility | Bhadra et al. (2018) | 26.2 | 12.0 |
| SA group 2 | Distribution parameter (CTDD) at first occurrence in the sequence using amino acids group 2 (R,K,Q,E,D,N) clustered by solvent accessibility | Bhadra et al. (2018) | 27.2 | 6.9 |
| SA group 3 | Distribution parameter (CTDD) at first occurrence in the sequence using amino acids group 3 (M,S,P,T,H,Y) clustered by solvent accessibility | Bhadra et al. (2018) | 100.0 | 8.4 |
| FET group 1 | Distribution parameter (CTDD) at first occurrence in the sequence using amino acids group 1 (I,L,V,W,A,G,T,M) clustered by the free energy to transfer from water to lipophilic phase | This study | 64.2 | 6.1 |
| FET group 2 | Distribution parameter (CTDD) at first occurrence in the sequence using amino acids group 2 (F,Y,S,Q,C,N) clustered by the free energy to transfer from water to lipophilic phase | This study | 16.9 | 6.1 |
| FET group 3 | Distribution parameter (CTDD) at first occurrence in the sequence using amino acids group 3 (P,H,K,E,D,R) clustered by the free energy to transfer from water to lipophilic phase | This study | 23.3 | 7.5 |
| Tiny res. % | Percent of residues of tiny amino acids (A + C + G + S + T) | Osorio et al. (2015) | 20.6 | 10.6 |
| Small res. % | Percent of residues of small amino acids  (A + B + C + D + G + N + P + S + T + V) | Osorio et al. (2015) | 14.4 | 17.7 |
| Aliphatic res. % | Percent of residues of aliphatic amino acids (A + I + L + V) | Osorio et al. (2015) | 16.3 | 6.0 |
| Aromatic res. % | Percent of residues of aromatic amino acids (F + H + W + Y) | Osorio et al. (2015) | 13.1 | 7.6 |
| Non-polar res. % | Percent of residues of non-polar amino acids  (A + C + F + G + I + L + M + P + V + W + Y) | Osorio et al. (2015) | 13.8 | 18.6 |
| Polar res. % | Percent of residues of polar amino acids (D + E + H + K + N + Q + R + S + T + Z) | Osorio et al. (2015) | 13.9 | 16.2 |
| Charged res. % | Percent of residues of charged amino acids (B + D + E + H + K + R + Z) | Osorio et al. (2015) | 12.6 | 9.6 |
| Basic res. % | Percent of residues of basic amino acids (H + K + R) | Osorio et al. (2015) | 21.8 | 25.9 |
| Acidic res. % | Percent of residues of acidic amino acids (B + D + E + Z); | Osorio et al. (2015) | 31.9 | 79.0 |
| Peptide charge | Peptide charge at pH 7.0 using "EMBOSS" pk-scale | EMBOSS | 34.5 | 88.4 |
| Isoelectric point | Peptide isoelectric point using "EMBOSS" pk-scale | Bjellqvist et al. (1994) | 29.9 | 42.8 |
| Aliphatic index | Relative volume occupied by aliphatic side chains (A, V, I, and L) in the peptide chain | Ikai (1980) | 17.5 | 7.9 |
| Instability index | Stability of a protein based on its dipeptide composition, it is used to determine whether the peptide will be stable in a test tube. | Guruprasad et al. (1990) | 20.8 | 10.3 |
| Boman index | Sum of the solubility values for all residues in a sequence, it might give an overall estimate of the potential of a peptide to bind to membranes or other proteins as receptors, to normalize it is divided by the number of residues | Boman (2003) | 18.4 | 21.0 |
| Hydrophobicity | Peptide's hydrophobicity using "KyteDoolittle" scale | Kyte and Doolitle (1982) | 15.7 | 11.0 |
| H-moment | Quantitative measure of the amphiphilicity perpendicular to the axis of any periodic peptide structure, such as the alpha-helix or beta-sheet using angle of 100º and a window of 11 residues | Eisenberg et al. (1984) | 24.0 | 21.8 |

^a^ Variable importance is a measure that refers to the frequency a given variable is used by the trees in the random forest

**REFERENCES:**

Bhadra P., Yan J., Li J. *et al.* AmPEP: Sequence-based prediction of antimicrobial peptides using distribution patterns of amino acid properties and random forest. *Sci Rep* 2018, 8: 1697. https://doi.org/10.1038/s41598-018-19752-w

Bjellqvist B., Basse B., Olsen E. and Celis J.E. Reference points for comparisons of two-dimensional maps of proteins from different human cell types defined in a pH scale where isoelectric points correlate with polypeptide compositions. Electrophoresis 1994, 15: 529-539.

Boman H.G. Antibacterial peptides: basic facts and emerging concepts. Journal of Internal Medicine 2003, 254(3): 197-215.

Eisenberg D., Weiss R.M., Terwilliger T.C. The hydrophobic moment detects periodicity in protein hydrophobicity. Proceedings of the National Academy of Sciences 1984, 81(1): 140-144.

EMBOSS data are from http://emboss.sourceforge.net/apps/release/5.0/emboss/apps/iep.html.

Guruprasad K., Reddy B.V., Pandit M.W. Correlation between stability of a protein and its dipeptide composition: a novel approach for predicting in vivo stability of a protein from its primary sequence. Protein Engineering 1990, 4 (2): 155–61.

Ikai A.J. Thermostability and aliphatic index of globular proteins. Journal of Biochemistry 1980, 88: 1895-1898.

Kyte J., Doolittle R.F. Hydropathicity. J. Mol. Biol. 157:105-132(1982).

Osorio D., Rondon-Villarreal P., Torres R. Peptides: A Package for Data Mining of Antimicrobial Peptides. The R Journal 2015, 7(1): 4-14.
