# Supplementary material for: Macrel: antimicrobial peptide screening in genomes and metagenomes
Source: PeerJ. 2020 Dec 18;8:e10555. doi: 10.7717/peerj.10555 (PMC7751412; doi:10.7717/peerj.10555)
Supplement: Supplemental Information 2 [file peerj-08-10555-s002.docx]

**Supplementary Table S2.** Datasets used in training and testing of classifiers implemented in Macrel.

| **Dataset** | **Positives** | **Negatives** | **Neg. : Pos.** | **Positives Length distribution (%)** | | | | | **Negatives Length distribution (%)** | | | | | **Overlap¹** | **Source** | **Experiment** |
| --- | --- | --- | --- | --- | --- | --- | --- | --- | --- | --- | --- | --- | --- | --- | --- | --- |
|  |  |  |  | **0-25** | **25-50** | **50-75** | **75-100** | **+** | **0-25** | **25-50** | **50-75** | **75-100** | **+** |  |  |  |
| Trainingset_AMPs_1_1 | 1197 | 1197 | 1 | 36.3 | 39.1 | 10.9 | 4.8 | 8.9 | 1.5 | 3.0 | 5.8 | 9.9 | 79.7 | 1_5,1_10,1_20,1_30,1_40,1_50 | Bhadra et al. (2018) | Homology |
| Trainingset_AMPs_1_5 | 1197 | 6000 | 5 |  |  |  |  |  | 1.2 | 2.1 | 5.4 | 10.0 | 81.3 |  | Bhadra et al. (2018) |  |
| Trainingset_AMPs_1_10 | 1197 | 12000 | 10 |  |  |  |  |  | 1.3 | 2.3 | 5.5 | 9.6 | 81.3 |  | Bhadra et al. (2018) |  |
| Trainingset_AMPs_1_20 | 1197 | 24000 | 20 |  |  |  |  |  | 1.2 | 2.1 | 5.8 | 9.9 | 80.9 |  | Bhadra et al. (2018) |  |
| Trainingset_AMPs_1_30 | 1197 | 36000 | 30 |  |  |  |  |  | 1.3 | 2.2 | 5.7 | 9.9 | 80.8 |  | Bhadra et al. (2018) |  |
| Trainingset_AMPs_1_40 | 1197 | 48000 | 40 |  |  |  |  |  | 1.3 | 2.1 | 5.6 | 10 | 80.9 |  | Bhadra et al. (2018) |  |
| Trainingset_AMPs_1_50 | 1197 | 60000 | 50 |  |  |  |  |  | 1.4 | 2.2 | 5.6 | 10.0 | 80.8 |  | Bhadra et al. (2018) |  |
| Testingset_AMPs_1_1 | 500 | 500 | 1 | 34.4 | 41.6 | 8.4 | 5.6 | 10 | 0.6 | 2 | 6.2 | 11.4 | 79.8 | None | Bhadra et al. (2018) |  |
| AMP.test | 920 | 920 | 1 | 44.7 | 52 | 3.3 | - | - | 10.6 | 3.7 | 19.4 | 66.3 | - | AMP.train | Xiao et al. (2013) | Macrel model |
| AMP.train_ bench | 1476 | 2405 | 2 | 48.3 | 42.9 | 5.4 | 3.3 | 0.1 | 10.2 | 11.2 | 29 | 49.6 | - | None | Xiao et al. (2013) |  |
| AMP.train | 3268 | 165138 | 50 | 37.5 | 40.8 | 8.7 | 3.7 | 9.3 | 1.5 | 2.5 | 5.6 | 10.1 | 80.3 | AMP.test | Bhadra et al. (2018) |  |
| Hemo.train | 442 | 442 | 1 | 76.0 | 23.3 | 0.4 | 0.3 | - | 74.7 | 22.4 | 2.2 | 0.7 | - | None | Chaudhary et al. (2016) |  |
| Hemo.test | 110 | 110 | 1 | 72.7 | 27.3 | - | - | - | 78.2 | 19.1 | 1.8 | 0.9 | - | None | Chaudhary et al. (2016) |  |

¹ Overlap in terms of homologous peptides
